# Deficits of Tactile Passive Perception Acuity in Patients With Schizophrenia

**DOI:** 10.3389/fpsyt.2020.519248

**Published:** 2020-10-27

**Authors:** Dan Liu, Hong Zhen Fan, Wen Xuan Zhao, Yun Hui Wang, Dong Li, Jing Long Wu, Tian Yi Yan, Shu Ping Tan

**Affiliations:** ^1^Huilongguan College of Clinical Medicine, Peking University, Beijing Huilongguan Hospital, Beijing, China; ^2^School of Mechatronical Engineering, Intelligent Robotics Institute, Beijing Institute of Technology, Beijing, China; ^3^School of Life Science, Beijing Institute of Technology, Beijing, China

**Keywords:** schizophrenia, tactile passive perception acuity, angle discrimination thresholds, cognition, working memory

## Abstract

**Background:** Scarce literature has yet to characterize the tactile discrimination capability as well as the underlying mechanism of tactile deficits in psychotic disorder. In particular, very little is known regarding the tactile perception acuity in schizophrenia.

**Methods:** A total of 131 clinically stable patients with schizophrenia (SCZ) and 79 healthy control (HC) volunteers were enrolled in the study. All the participants were tested on a tactile stimulus device which could quantify the tactile discrimination capability with right index finger scanned over the angles via the passive finger-movement apparatus. The MATRICS Consensus Cognitive Battery (MCCB) was adapted to assess the neurocognition of the participants. Correlation analysis and multivariate linear regression analysis were performed to investigate the relationship between tactile perception performance and neurocognitive function.

**Results:** It was discovered that there existed a significant deficits in the tactile passive perception acuity (i.e., tactile angle discrimination threshold) in patients with schizophrenia compared with their healthy controls (F _(3, 206)_ = 11.458, *P* = 0.001,partial η^2^ = 0.053). The MCCB total score and its six domains were significantly lower in SCZ patients than those in HCs (all *p* < 0.001). In the SCZ group, the composite score of the MCCB (*r* = −0.312, *P* < 0.001) and domains of neurocognition including speed of processing (*r* = −0.191, *P* = 0.031), attention/vigilance (*r* = −0.177, *P* = 0.047), working memory (*r* = −0.316, *P* < 0.001), verbal learning (*r* = − 0.332, *P* < 0.001), visual learning (*r* = −0.260, *P* = 0.004), and reasoning and problem solving (*r* = −0.209, *P* = 0.018) showed significant negative correlations with the tactile angle discrimination threshold. Multivariate linear regression analysis revealed that neurocognition impairment, especially the decline of working memory (B = −0.312, *P* < 0.001),underpin the tactile perception discrimination deficits in patients with SCZ.

**Conclusion:** To the best of our knowledge, this is the first study to unravel the deficits of tactile passive perception acuity and its underlying neurocognition basis in patients with SCZ. This finding adds novel evidence to the subtle variation in haptic discrimination skills in schizophrenia which contributes to a more comprehensive understanding of the sensory profiles of this disorder.

## Introduction

Schizophrenia (SCZ) is a major psychiatric disorder characterized by a wide range of neurocognitive deficits (1, 2) and somatosensory disturbances (3). Patients with SCZ exhibit reduced sensory gating in the somatosensory domain (4) and attenuated pain sensitivity (5, 6). Somatosensory disturbances precede the onset of psychosis and can be found in drug-naive patients (7). In addition, lack of somatosensory attenuation is related to aberrant sense of agency, which may spawn a misattribution of self-generated actions to external sources, thus contributing to development of psychotic symptoms, such as auditory hallucinations and delusions of control (8). For instance, some studies showed that the classical rubber hand illusion (RHI) (9–11), in which individuals misattribute tactile sensations “felt” by their real hand hidden from view to a rubber prosthetic hand that they “see” being tactilely stimulated in synchrony, was enhanced in SCZ patients compared with normal controls (12). Other studies using a new induction procedure of the RHI relying on tactile expectation rather than proper visuo-tactile stimulation further demonstrated that schizophrenia individuals exhibited a weaker sense of ownership over the rubber hand than did healthy controls (13). Moreover, decreased spatial acuity and impaired perceived intensity in the processing of tactile stimuli are observed in the biological relatives of SCZ patients (14), and the tactile extinction phenomenon, a repetitively-reported clinical sign of parietal lobe disease (15), has been found in schizophrenia patients (16). These findings suggest impaired tactile perception in SCZ patients and its potential “trait” characteristic.

Tactile perception involves the complementary interaction of the bottom-up peripheral sensory process and top-down central cognitive mechanisms for detection and sensation of objects and discrimination and evaluation of their size, shapes, and surface characteristics (17, 18). It is well-documented that tactile perceptual skills decline with aging (17, 19–23), and age seems to have a stronger influence on the perceptive-evaluative capacities (i.e., top-down processes, such as cortical neuronal tuning properties, lateral inhibition, attention, and working memory) than on the stimulus-driven sensitivity (i.e., bottom-up processes, such as skin characteristic and receptor density) (17). In addition, studies have shown that geometric properties of objects, which are generally composed of shape and size, typically necessitate the cutaneous and kinesthetic input to serve the haptic processing (24). In other words, shape discrimination of objects under certain circumstances can be revealed by skin indentation because they fit within the fingertip, whereas shape perception of objects with contours that extend beyond the fingertip scale frequently relies on the kinesthetic inputs. Moreover, albeit Gibson et al. (25) posited that active touch deemed as active exploration and manipulation of surfaces and objects with our hand (26) is more likely to yield objective percepts and veridical perception, whereas passive touch referred to stimulating the stationary finger or hand with a moving or static external stimulus (26) tends toward subjectivity (27), neuroimaging evidence suggests that somatosensory regions activated by active shape discrimination are highly in concordance with passive shape discrimination (28). Furthermore, it is of great indispensability to be aware of the extent to which the cutaneous system is limited by its ability to resolve spatial and temporal details presented on the skin. Notably, accumulating evidence indicates that tactile spatial acuity varies significantly across the body surface, and the fingertip is far more sensitive than the palm and other superficial parts of the body, such as nose, forehead, belly, thigh, or sole (24).

Studies with regard to somatosensory discrimination, such as discrimination between different textures, sizes, and shapes of objects; two-point tactile discrimination; stereognosis; and graphesthesia are scarce, reflecting a critical gap since the somatosensory system serves as an important foundation for many aspects of human development, including fine motor skills (e.g., grasp) and social and communication skills (29).Of striking note, impaired sensory discrimination task performance has been demonstrated in autism spectrum disorder (ASD) patients compared with typically developing (TD) controls (30–33); for example, high-functioning people with ASD exhibited defective right-handed graphesthesia (34) and poorer stereognosis performances (35) compared with TD individuals. Originated from severe neurodevelopmental disorders, sensory perception dysfunction, which is characterized by failing to perceive and integrate multisensory modality information, is one of the most vital characteristics shared by SCZ and ASD. This inability to process multisensory system information may lead to misinterpretation of social information, therefore contributing to the social cognition deficits across these two mental disorders.

Tactile spatial discrimination is deemed as one of the main manual learning and memory skills in humans. Studies have demonstrated that abnormal somatosensory information processing contributes to the functional decline of tactile shape discrimination in patients with mild cognitive impairments and Alzheimer's disease (36). The somatosensory system is a diverse sensory system that comprises the receptors and processing centers, which make up sensory modalities (37, 38), and the skin, as the body's largest organ, is the first point of contact with the environment and encodes information from many different sources in addition to providing a sense of boundary or self and non-self discrimination; thus, the skin is a backdrop to all perceptual experiences (39). However, to the best of our knowledge, tactile angle acuity discrimination in patients with SCZ has not yet been characterized, and its underlying neurocognitive basis remains poorly understood. With this above-mentioned consideration in mind, we hypothesized that (1) tactile angle discrimination thresholds would be significantly higher in SCZ patients than in their healthy counterparts, and that (2) well-accepted neurocognitive deficits in the SCZ [i.e., in whom impaired cognition is a hallmark (40)] are negatively associated with the tactile perception acuity, especially in certain domains of neurocognition, such as working memory.

## Materials and Methods

### Participants

This study enrolled 131 clinical stable patients with a diagnosis of schizophrenia or schizoaffective disorder (41) who were being treated with stable antipsychotic medication during the study period and 79 healthy controls without personal or family history of psychiatric illness assessed with the Mini-International Neuropsychiatric Interview (42, 43). All the patients were recruited from Beijing Huilongguan Hospital (Huilongguan College of Clinical Medicine, Peking University, Beijing, China) from November 1, 2017 to December 31, 2018, and healthy controls were volunteers in the surrounding communities. SCZ or schizoaffective disorder was diagnosed based on the Structured Clinical Interview for the criteria of the Diagnostic and Statistical Manual of Mental disorders, Fourth Edition (41). Inclusive criteria were as follows: (1) age between 18 and 60 years, (2) more than 6 years of full-time education to ensure that participants can understand task instructions, (3) normal or corrected-to-normal auditory and visual acuity to eliminate tactile dominance due to other modality defects, (4) sufficient command of the Mandarin Chinese language without dysarthria, and (5) right handedness measured by the Edinburgh Handedness Inventory (44). Participants with remarkable abnormality in motor or sensory systems and deep tendon reflexes, loss of tactile sensation or any unusual experiences with haptic input, mental retardation or neurological illness, severe depression or anxiety symptoms, alcohol/substance dependence or abuse, or presence of any physical disease that could impair tactile sensation, and pregnant or lactating women were excluded in this study. The study was approved by the Ethics Committee of Beijing Huilongguan Hospital and was conducted in line with the Declaration of Helsinki. All the participants provided written informed consent after full explanation of the study procedure. Study participants were given a certain monetary inconvenience allowance for their full participation.

### Clinical Assessment

Clinical characteristics of SCZ patients were collected by two experienced psychiatrists in a direct interview. When it was difficult to obtain clinical information via the patients *per se*, medical records, as well as the information provided by their caregivers, would be combined. Socio-demographic and clinical variables, including duration of the psychosis and current pharmacological treatment, were assessed. The dose of antipsychotics was converted to the chlorpromazine equivalent (45). The Positive and Negative Symptom Scale (PANSS) (46) was adopted to assess the severity of the psychotic symptoms of SCZ by four attending psychiatrists, with an accepted inter-rater consistency (Kappa value, 0.85). In addition, the severity of affective symptoms was assessed using the Calgary Depression Scale for Schizophrenia (CDSS) (47, 48), with CDSS score of ≥ 6 points as significant depression in SCZ. The CDSS evaluates depression, hopelessness, self-depreciation, guilty ideas of references, pathological guilt, morning depression, early wakening, suicide, and observed depression. Each item is scored from 0 to 3, with a higher score indicating more severe depression. Healthy controls were assessed using the 9-item Patient Health Questionnaire (PHQ-9) (49) and the Generalized Anxiety Disorder (GAD-7) scale (50) (≥5 points as the cut-off points for both scales) to screen concomitant serious depression and anxiety symptoms.

### Neurocognition Measurement

Neurocognitive function of the participants was assessed using the Measurement and Treatment Research to Improve Cognition in Schizophrenia (MATRICS) Consensus Cognitive Battery (MCCB) (51), which derives from the National Institute of Mental Health's MATRICS, and was initially used as a primary outcome measure for clinical trials of cognitive assessment in SCZ. The MCCB neuropsychological measure consists of 10 tests which converged into seven cognitive domains: (1) Speed of Processing, which is measured with the Trail Making Test Part A, the Brief Assessment of Cognition in Schizophrenia-symbol coding, and Category Fluency Test-Animal Naming and mainly reflects the visual scanning speed or visuo-motor tracking and verbal processing speed; (2) Attention/vigilance, which is depicted with Continuous Performance Test-Identical Pairs; (3) Working Memory, which is assessed with Wechsler Memory Scale-III-Spatial Span (nonverbal working memory), and Digit Sequencing (verbal working memory); (4) Verbal Learning, which is portrayed with the Hopkins Verbal Learning Test-Revised; (5) Visual Learning, which is measured with the Brief Visuospatial Memory Test-R; (6) Reasoning and Problem Solving, which is assessed with Maze Test (Neuropsychological Assessment Battery-Mazes), a reflection of planning and executive function; and (7) Social cognition, which is measured with the Mayer-Salovey-Caruso Emotional Intelligence Test. MCCB performances were converted to T scores (mean, 50; standard deviation, 10) based on the raw scores adjusted for age, sex, and years of education, with higher scores indicating better neurocognitive performance. The MCCB cognition assessment was administrated by two trained clinical psychologists who had over 5 years of experience in psychometric testing, with a satisfactory inter-rater consistency (Intraclass Correlation Coefficient, 0.92).

## Task Paradigm and Procedure

The tactile angle discrimination apparatus via finger sensation was employed to detect the haptic perception acuity of the participants in the current study, as previously described (36, 52). The tactile perception device is composed of an electric conveyer belt that moves the angles varying in size along the horizontal axis in the transverse plane. Within each trial, a pair of angles with one reference angle and another comparison angle were successively presented to the participant to make an angle-comparison judgment (there were three alternative choices, i.e., “great than,” “smaller than,” and “equal to,” after the presence of the second angle). The least reference angle was 60°, and the predetermined comparison angles were larger than the reference angle by 4°, 8°, 12°, 16°, 20°, 24°, 32°, and 50°. The angles were mounted below an imaginary bisector such that two raised arms (i.e., 0.5 mm) were symmetrically horizontally placed above, with the apex of the angles always pointing to the right. In other words, all of the angles moved in the fixed parallel plane over a 40.0-mm square base, and the angle size was set as the sole difference. The electric conveyer belt movement was confined to a maximum range of 200.0 mm, and the movement speed of the angles was maintained at 5.0 mm/s. All angles were moved from the end-points toward the apex. A pseudorandom order was used to present the reference angle and the comparison angle to the participants. Participants were instructed to render their right hand immobile so that only their right index finger was able to contact with the angles, and the participants were asked to avoid using any visual feedback during the task as best as possible. Each participant underwent at least 10 practice trials prior to the start of the test. Subsequently, each pair of angles was presented 10 times in a pseudorandom order, and each participant completed 80 angle discrimination trials.

### Statistical Analysis

Demographics of participants, including age and years of education, were compared using a Student's *t*-test, and chi-squared test was used for categorical variables, such as sex. The duration of psychosis, antipsychotics, and the PANSS scale scores were described, and analysis of covariance was employed for the comparison of MCCB scores between groups, with sex and years of education as the covariates. A two-way of analysis of variance (ANOVA) was performed to investigate the differences in the tactile angle discrimination threshold between the two groups, with group (patient vs. control) as an inter-subject factor, sex (male vs. female) as a within-subject factor, and age and years of education as covariates. Moreover, partial correlation analysis and multiple linear regression were performed to analyze the relationship between neurocognition function and angle discrimination threshold. All statistical analyses were performed using SPSS for Windows version 20.0 (IBM Corp, Armonk, NY, USA). For all tests, significance was set at *P*-value **<** 0.05.

## Results

### Demographic, Clinical, and Neurocognitive Characteristics of Participants

A total of 131 schizophrenia patients (the SCZ group) and 79 healthy controls (HCs) participants (the HC group) were included in the current study. There was a higher proportion of males in SCZ group than in HC group (54.96% vs. 36.71%; χ^2^ = 6.975, *P* = 0.008). Moreover, schizophrenia patients had lower years of education than their healthy counterparts (13.09 ± 2.95 vs. 14.24 ± 3.01; *t* = 2.714, *P* = 0.007). No significant age difference was observed between groups (*t* = 1.698, *P* = 0.091). Both schizophrenia patients (CDSS: 2.95 ± 3. 72) and healthy controls (PHQ-9: 1.68 ± 2.68 vs. GAD-7: 1.50 ± 2.31) did not show notable affective symptoms. All the participants were right-handed. As for the neurocognition assessment, SCZ patients showed significant poorer performances in the domains of processing speed, attention/vigilance, working memory, verbal learning, visual learning, reasoning and problem solving, and the composite scores than did HCs (Table 1).

**Table 1:** Demographic and clinical characteristics.

	**SCZ (*n* = 131)**	**HC (*n* = 79)**	**t/χ^2^**	***P***
Age (mean/SD)	44.38 (12.34)	41.67 (10.46)	1.698	0.091
Years of education (years, mean/SD)	13.09 (2.95)	14.24 (3.01)	2.714	0.007
Sex, *n* (%)			6.975	0.008
Male	72 (54.96)	29 (36.71)		
Female	59 (45.04)	50 (63.29)		
Handedness, Right, *n* (%)	131 (100)	79 (100)		
Duration of illness (years, mean/SD)	16.87 (11.97)			
PANSS (mean ± SD)				
Positive Scores	14.06 (6.10)			
Negative Scores	21.08 (5.90)			
General Psychopathology Scores	30.89 (8.56)			
Total Scores	66.03 (15.16)			
CDSS	2.95 (3.72)			
PHQ-9		1.68 (2.68)		
GAD-7		1.50 (2.31)		
Antipsychotics, *n* (%)				
Typical	6 (4.58)			
Atypical	117 (89.31)			
Combination of typical and atypical	8 (6.11)			
CPZ equivalent (mean/SD, mg)	565.79 (227.73)			
MCCB measure (mean/SD)				
Speed of processing (TMT-A/BACS/CFT) ^a^	43.58 (9.71)	57.53 (7.62)	103.513	<0.001
Attention/Vigilance (CPT-IP) ^a^	44.31 (9.27)	56.24 (7.44)	79.048	<0.001
Working memory (SS/DS) ^a^	46.05 (11.92)	57.08 (7.87)	42.470	<0.001
Verbal learning (HVLT) ^a^	46.04 (11.30)	56.76 (8.06)	45.066	<0.001
Visual learning (BVMT) ^a^	44.26 (10.17)	52.62 (8.97)	29.475	<0.001
Executive function (NAB-Maze) ^a^	44.02 (0.99)	52.62 (8.97)	53.929	<0.001
Social cognition (MSCEIT) ^a^	47.03 (0.28)	50.16 (10.23)	2.491	0.116
Composite Score^a^	43.55 (9.88)	56.51 (6.89)	89.251	<0.001

*CPZ equivalent:chlorpromazine equivalent. PANSS, Positive and Negative Symptoms Scale; CDSS, Calgary Depression Scale for Schizophrenia; PHQ-9, 9-item Patient Health Questionnaire; GAD-7, 7-item Generalized Anxiety Disorder Scale; MCCB, the MATRICS Consensus Cognitive Battery; TMT-A, Trail Making Test Part A; BASC, the Brief Assessment of Cognition in Schizophrenia (BASC) -symbol coding; CFT, Category Fluency Test-Animal Naming; CPT-IP, Continuous Performance Test-Identical Pairs; SS, Spatial Span; DS, Digit Sequencing; HVLT, Hopkins Verbal Learning Test; BVMT, Brief Visuospatial Memory Test; NAB-Maze, Neuropsychological Assessment Battery-Mazes,NAB-MAZES; MSCEIT, Mayer-Salovey-Caruso Emotional Intelligence Test. ^a^ANOCOVA was employed to compared the differences of MCCB between groups, with gender and years of education as covariates, and statistic F value were shown*.

### Tactile Perception Acuity (Angle Discrimination Threshold) Deficits in Schizophrenia

**Figure 1:**
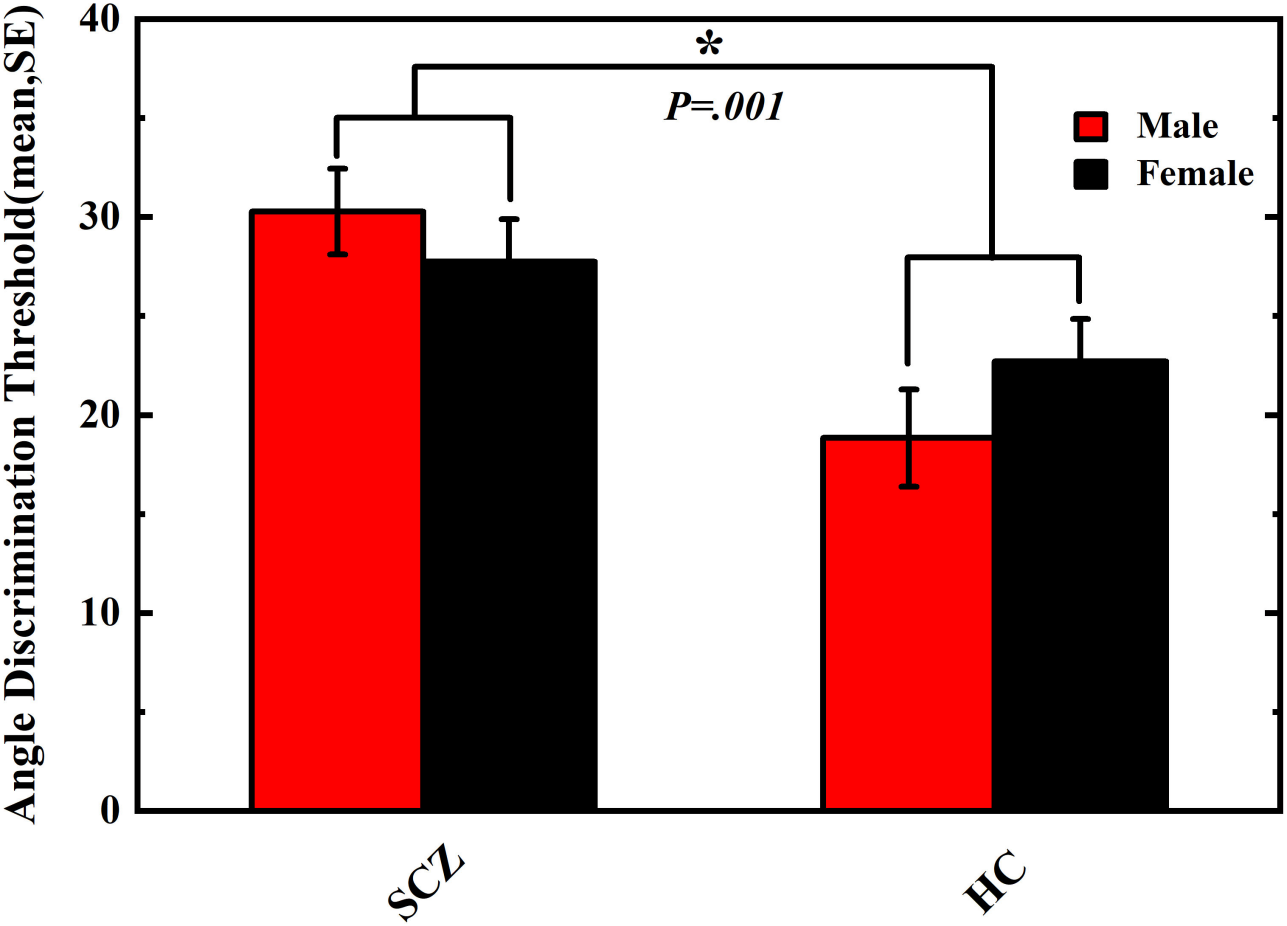
Angle discrimination threshold of schizophrenia patients and healthy controls. Comparison of angle discrimination thresholds in male and female participants between schizophrenia and healthy control. Vertical error bar represents standard error of the mean. Asterisk means *P* < 0.05.The difference of tactile angle discrimination threshold between SCZ group and HC group was significant (*P* = 0.001).

In our study, the results of the tactile perception trial reported by the participants were logistically transformed to the angle discrimination threshold via plotting a logistic curve function of the angular differences between the comparison and reference angles; the obtained angle discrimination threshold indicates the minimum angle size that participants could distinguish, and the smaller value of angle discrimination threshold indicates the better capability of tactile perception to distinguish the angle differences. Results of the tactile perception task performance are shown in Figure 1. The two-way ANOVA revealed a significant main effect of group (F _(3, 206)_ = 11.458, *P* = 0.001, partial η^2^ = 0.053), but no significant main effect of sex (F _(3, 206)_ = 0.053, *P* = 0.818, partial η^2^ = 0.000) or group^*^sex interaction (F _(3, 206)_ = 1.676, *P* = 0.197, partial η^2^ = 0.008) was observed. Male (30.66 ± 20.09) and female (27.22 ± 16.77) SCZ patients exhibited poorer performances in angle discrimination threshold than male (18.85 ± 13.20) and female (22.70 ± 15.19) HCs, respectively, indicating deficits in the tactile perception acuity in SCZ patients.

### Correlation Analysis Between Neurocognition Function, Clinical Symptoms, and Angle Discrimination Threshold

**Figure 2:**
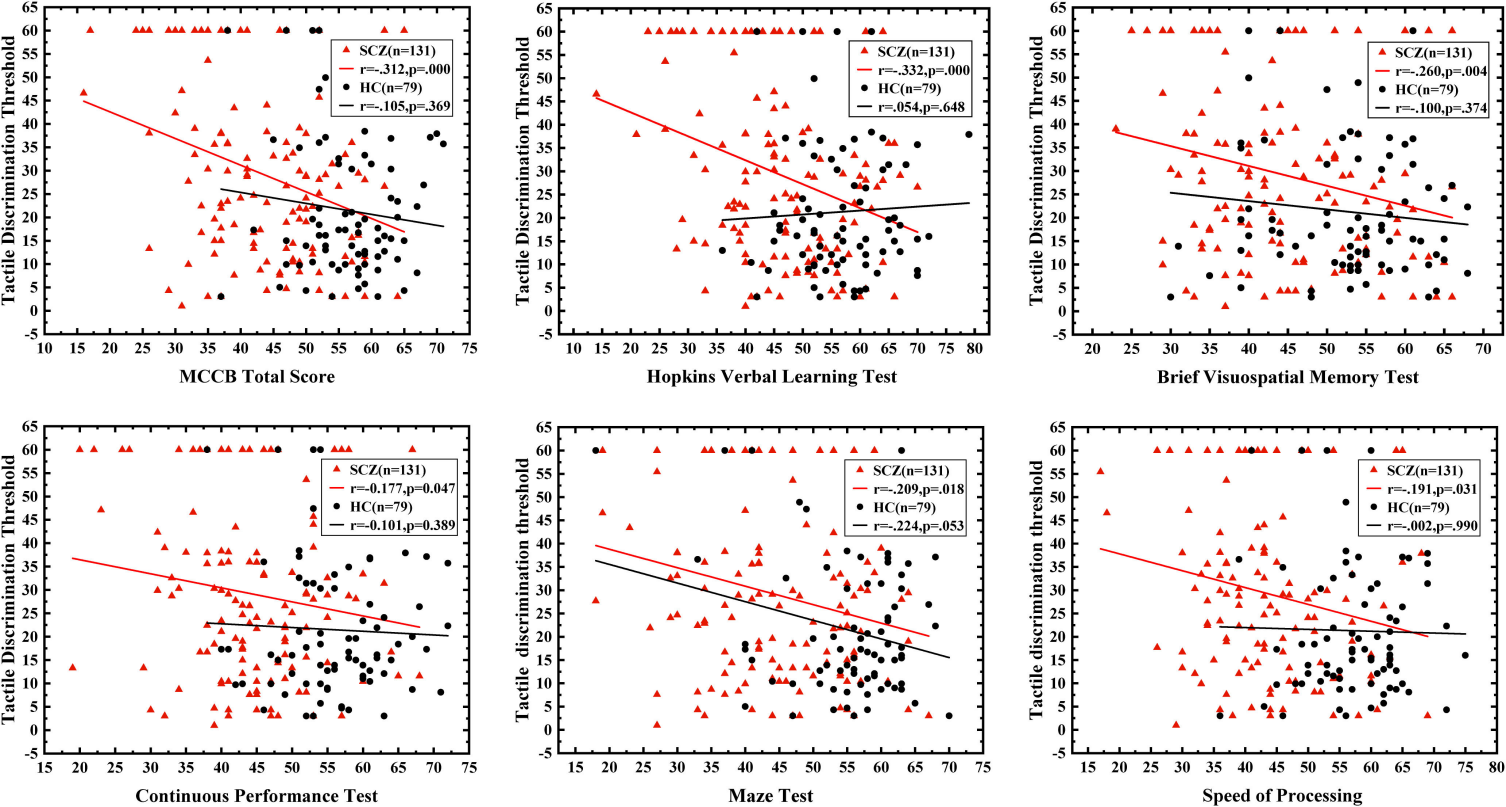
The correlation between neurocognition function and tactile angle discrimination threshold. The red triangle represents schizophrenic patients, and the black circle refers to healthy controls. The correlation between neurocognition domains and tactile angle discrimination threshold of the two groups was shown. All the MCCB domain except for the social cognition were negatively associated with tactile angle discrimination threshold, and the correlation was only significant in patients group.

In the SCZ group, domains of neurocognition including speed of processing (*r* = −0.191, P = 0.031), attention/vigilance (*r* = −0.177, *P* = 0.047), working memory (*r* = −0.316, *P* < 0.001), verbal learning (*r* = −0.332, *P* < 0.001), visual learning (*r* = −0.260, P = 0.004), and reasoning and problem solving (*r* = −0.209, *P* = 0.018) showed significant negative correlations with the tactile angle discrimination threshold. In addition, the composite score of the MCCB (*r* = −0.312, *P* < 0.001) was negatively correlated with the angle discrimination threshold as well (Figure 2), revealing that poorer neurocognition performance was associated with severer deficits in the angle discrimination threshold in schizophrenic patients. However, no significant correlation was observed between social cognition (*r* = −0.056, *P* = 0.530) and the angle discrimination threshold. Moreover, there was no relationships between clinical symptoms obtained using the PANSS positive (*r* = 0.123, *P* = 0.173), PANSS negative (*r* = 0.084, *P* = 0.352), PANSS general psychopathology (*r* = 0.097, *P* = 0.285), or PANSS total score (*r* = 0.137, *P* = 0.129) and tactile angle discrimination threshold. In the HC group, neurocognition domains of reasoning and problem solving were marginally negatively associated with the tactile angle discrimination threshold (*r* = −0.224, *P* = 0.053), and no correlation between the other domains of the MCCB or the MCCB total score and tactile angle discrimination threshold was observed (*r* = −0.002 to−0.110, all *P* > 0.05) (Figure 2).

### Multivariate Linear Regression Analysis of the Tactile Angle Discrimination Threshold

**Figure 3:**
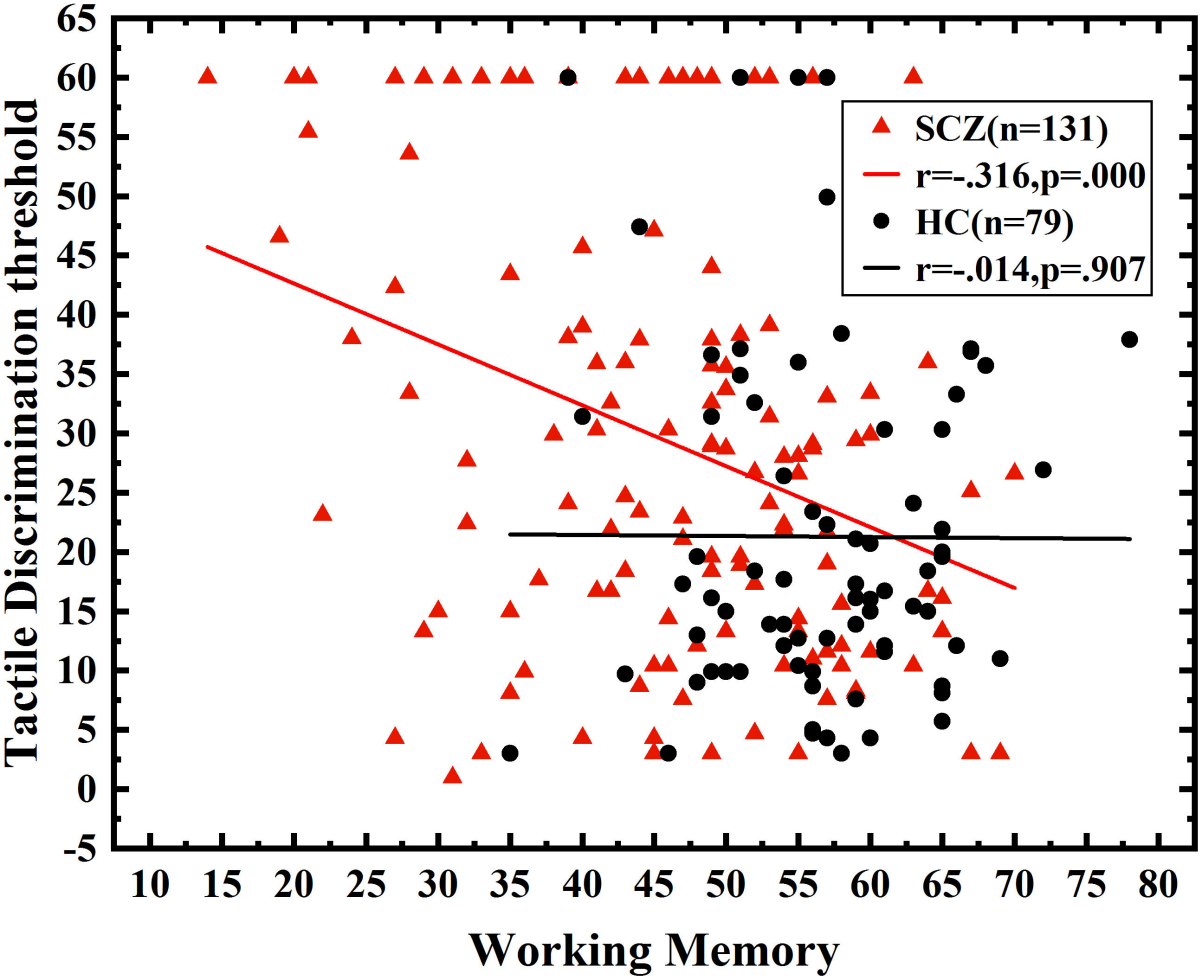
The correlation between working memory and angle discrimination threshold. Correlation analysis between working memory and tactile angle discrimination thresholds. The performance of working memory was negatively correlated with the angle discrimination threshold in SCZ (*r* = −0.316, *P* < 0.001).

A multivariate regression analysis was conducted to further assess the relationship between neurocognition domains and tactile angle discrimination thresholds in SCZ, with the angle discrimination threshold as a dependent variable and seven cognition domains as independent variables. The neurocognition domains explained 9.8% of the variance (R^2^ = 0.098, *P* < 0.001; adjusted R^2^ = 0.090, *P* < 0.001), and the working memory had a significant negative predictive effect on the deficits in the angle discrimination threshold (B = −0.312, *P* < 0.001) in SCZ, while neither of the other six domains made a contribution to the model (Figure 3, Table 2).

**Table 2:** The multivariate linear regression (Enter) model of tactile angle discrimination threshold.

	**Coefficients**		***t***	***P***	**95%CI for B**
	**B**	**Std.Error**			
Speed of Processing	0.143	0.242	1.158	0.249	−0.199~0.760
Attention/Vigilance	0.017	0.214	0.155	0.877	−0.390~0.457
Working memory	**−0.312**	**0.134**	**−3.691**	** <0.001**	**−0.757~−0.229**
Verbal learning	−0.204	0.196	−1.641	0.103	−0.710~0.067
Visual learning	−0.032	0.232	−0.249	0.804	−0.517~0.401
Executive function	−0.110	0.178	−1.054	0.294	−0.540~0.165
Social cognition	0.028	0.166	0.301	0.764	−0.279~0.379

*Standardized beta coefficients were displayed. Working memory had significant negative predict effect to the deficits in tactile angle discrimination threshold in SCZ (B = −0.312, P < 0.001)*.

## Discussion

While a considerable amount of studies have investigated somatosensory disturbance and motor incoherence in SCZ, much less is known about fine haptic skills of this population. Accordingly, we investigated the tactile angle discrimination capability in schizophrenia patients with a special angle discrimination device that could quantify tactile perception acuity. The current study revealed significant deficits in the angle discrimination threshold capacities in schizophrenia patients compared with age-matched HCs. Moreover, we found that neurocognitive function, especially working memory, was negatively related to the angle discrimination threshold performance, suggesting that neurocognitive impairment underlies the deficits in tactile perception acuity (measured as tactile angle discrimination threshold) in SCZ. The findings shed new light on the underlying characteristics of the somatosensory disorder in patients with SCZ.

Our angle discrimination threshold paradigm with a pair of angles randomly presented to participants via passive touch without visual feedback includes the following possible procedures: (i) encoding the first angle, extracting the relevant parameter, and storing the parameter value in memory; (ii) encoding the second angle, extracting the relevant parameter, and comparing the second angle parameter with the memory of the first angle parameter; and (iii) making a decision based on the outcome of the comparison (53). Although neural mechanisms underlying deficits in haptic perception acuity and its relationship with working memory impairment remain unclear, we speculate that the tactile angle discrimination procedure activates a widely distributed cerebral network that mainly includes areas involved in the initial processing of skin indentations (i.e., primary and secondary somatosensory cortex) (54, 55), higher-order areas for computation and elaborate reconstruction of shapes [i.e., anterior part of the intraparietal sulcus (25)], and the prefrontal cortex (56),which is activated in tactile working memory processing. In line with the pre-existing findings showing that working memory contributes to the performance of somatosensory discrimination (53), our study revealed that SCZ patients (but not healthy controls) with more severe working memory impairment exhibited poorer tactile angle discrimination ability. The findings indicate that brain regions involved in basic neurocognitive processing underpinned by a more cohesive pattern of brain activation are activated in schizophrenic patients, but not in healthy controls, and the cognitive load is significantly increased in patients with SCZ for better identification of the magnitude between a comparison angle and a reference angle.

Although the pathophysiology of SCZ remains to be determined, accumulating evidences have demonstrated elevated presynaptic dopamine function in the striatum (57) as well as the neuroanatomical and electrophysiological alterations in the medial temporal lobe, including the hippocampus and different areas of the prefrontal cortex (58), in SCZ patients. And current findings on genetic and environmental causes of SCZ have linked this disorder to abnormal neurodevelopment (59, 60). Human brain development lasts more than two decades, from embryonic patterning *in utero* to synaptic pruning in adolescence (61), and there are several sensitive windows when even the most subtle variation in the organization of brain circuits could contribute to functional alterations that persist throughout one's lifetime (62); some functional alterations are linked to primary functions, such as vision and touch, and others are associated with more complex tasks that involve cognitive experiences, such as language acquisition or specific social behaviors (63).

Similar to neurons in the central nervous system, epithelial tissues, such as tactile corpuscles in the skin's dermal papillae that function in tactile sensation, derive from the ectoderm. It is presumable that tactile perception acuity deficits are associated with neurocognitive impairment caused by neurodevelopmental abnormality from phylogenetic and ontogenetic perspectives. Notably, the duration of disease and PANSS scores, which could partly reflect the severity of the disease, are not associated with the tactile perception acuity in SCZ, suggesting that subtle variations of haptic discrimination skills in SCZ are not linked to the course or severity of the disease, which in line with the previous studies showing that tactile perception disturbance precedes the onset of disease (7).Therefore, rather than as a “state” indicator that varies with the course of the disease, tactile perception acuity deficits in SCZ are more likely to be linked to the disease risk and promises to be used as an potential “trait” biomarker for assisting disease diagnoses and prediction of the efficacy and outcomes.

The present study has some strengths. To the best of our knowledge, few studies have focused on tactile perception acuity capability in SCZ, and this study is the first to investigate the tactile angle discrimination performance and its neurocognitive basis in SCZ. In addition, we used continuous measurements of angle discrimination thresholds to define tactile variations, which would otherwise be difficult to assess. Further, the sample size is sufficient for a behavioral paradigm study, further supporting the robustness of our findings. However, our study has some limitations. First, both visuo-spatial and somatic codes are involved in the tactile sensation (64), and the interaction between the visual and tactile modalities might influence haptic perception (65). However, in this study, we attempted to avoid visual feedback in the current tactile stimulus task, thus the effects of vision or visual attention on tactile discrimination differences between groups could be partly negligible. Second, tactile perceptual learning (66, 67) was not investigated in the current study. However, the randomized stimulus presentation helps to attenuate the learning effects, and perceptual learning involves relatively long-lasting changes rather than only a disposable practice in an organism's perceptual system to improve the ability of the system to respond to its environment (66). Thus, the differences between SCZ patients and healthy controls are not likely to be the results of learning effects. Third, the included participants were chronic schizophrenia patients, and the effects of drugs on the present findings cannot be ruled out. Further studies are needed to determine whether there are tactile perception acuity deficits in first-episode schizophrenia patients. Fourth, since our study was a cross-sectional study without longitudinal follow-up, it is difficult to derive causal relationships based on the present findings. Further longitudinal studies are needed to determine the generalizability of the present findings.

In conclusion, this study found tactile perception acuity deficits and their association with neurocognitive impairment in schizophrenia patients. This finding adds novel evidence to the subtle variation in haptic discrimination skills in schizophrenia which contributes to a more comprehensive understanding of the sensory profiles of this disorder. Future studies examining basic perception processing capacities and their relevance to underlying genetic and molecular mechanisms are needed to determine whether the angle discrimination threshold could be used as a “trait” biomarker in SCZ for assistance in the diagnosis of diseases or the prediction of treatment efficacy and outcomes.

## Data Availability Statement

The datasets analyzed in this article are not publicly available. Requests to access the datasets should be directed to Star950214@126.com.

## Ethics Statement

The studies involving human participants were reviewed and approved by Ethics Committee of Beijing Huilongguan Hospital. The patients/participants provided their written informed consent to participate in this study.

## Author Contributions

DLiu executed the analyses, interpreted the data, and wrote the first draft of the manuscript. ST and TY conceived and designed the study, supervised the project, and gave important suggestions for the revision of the manuscript. HF undertook the statistical analyses. WZ, YW, and DLi collected the clinical data and aided with angle discrimination trail. JW provided important technical support. All authors contributed to and have approved the final manuscript.

## Conflict of Interest

The authors declare that the research was conducted in the absence of any commercial or financial relationships that could be construed as a potential conflict of interest.
